# Design of dynamic genetic memory

**DOI:** 10.1049/iet-syb.2017.0021

**Published:** 2017-12-01

**Authors:** Yu‐Jia Hu, Chun‐Liang Lin, Wei‐Xian Li

**Affiliations:** ^1^ Department of Electrical Engineering National Chung Hsing University 402 Taichung Taiwan

**Keywords:** DRAM chips, genetic engineering, biocomputers, bioinformatics, equivalent circuits, RC circuits, dynamic genetic memory design, electronic systems, dynamic random access memory, modern silicon computer, biocomputer, bioinformation, binary logic, logical high level, logical low level, normalised form, genetic DRAM, modified electronic configuration, biological reaction, equivalent RC circuit, memory cell, fundamental functions, genetic toggle switch, data hold, biostorage module

## Abstract

In electronic systems, dynamic random access memory (DRAM) is one of the core modules in the modern silicon computer. As for a bio‐computer, one would need a mechanism for storage of bio‐information named ‘data’, which, in binary logic, has two levels, logical high and logical low, or in the normalised form, ‘1’ and ‘0’. This study proposes a possible genetic DRAM based on the modified electronic configuration, which uses the biological reaction to fulfil an equivalent RC circuit constituting a memory cell. The authors implement fundamental functions of the genetic DRAM by incorporating a genetic toggle switch for data hold. The results of simulation verify that the basic function can be used on a bio‐storage module for the future bio‐computer.

## 1 Introduction

Synthetic biology is an emerging research topic that develops artificial biological systems by investigating biomedical reaction in the cell [1, 2]. It combines knowledge of biological engineering, electrical engineering, and computer science, by measuring and observing how real biological tissue works, establishing mathematical models that characterise live cell's behaviour, and testing model differences. The synthetic biological circuit is a possible application of synthetic biology that uses biological discipline to perform equivalent logic functions in the electric circuits [3, 4]. Recently, a computer aided design tool for developing combinational gene logic circuits using the idea of constructing a variety of biobricks was developed [5]. Based on the previous achievements in this field, one would be able to apply the electrical engineering's principle to biological processes [6, 7]. In 2000, a pioneering work developed by Elowitz and Leibler [8] unveiled a genetic oscillator. In the same year, a genetic toggle switch was proposed by Gardner *et al.* [9]. Since then, an increasing number of genetic circuits such as genetic Boolean logic gates using the cis‐regulatory network were presented, and more complex genetic logic circuits like combinational genetic logic circuits and genetic sequential circuits appeared one after another [10, 11].

However, there is still a lack of concrete results developed in the area of synthetic biology as a basis for the future bio‐computer data storage [12, 13]. The von Neumann model describes a fundamental architecture for a digital computer with a processing unit which includes an arithmetic logic unit and several processor registers, a control unit containing an instruction register and program counter, a memory to store both of data and instructions, an external mass storage, and input and output mechanisms. Although previous research has built up a class of combinational gene circuits like bio‐register [14] towards the von Neumann computer, the technology of bio‐storage unit only receives limited attention [15, 16].

**Fig. 1:**
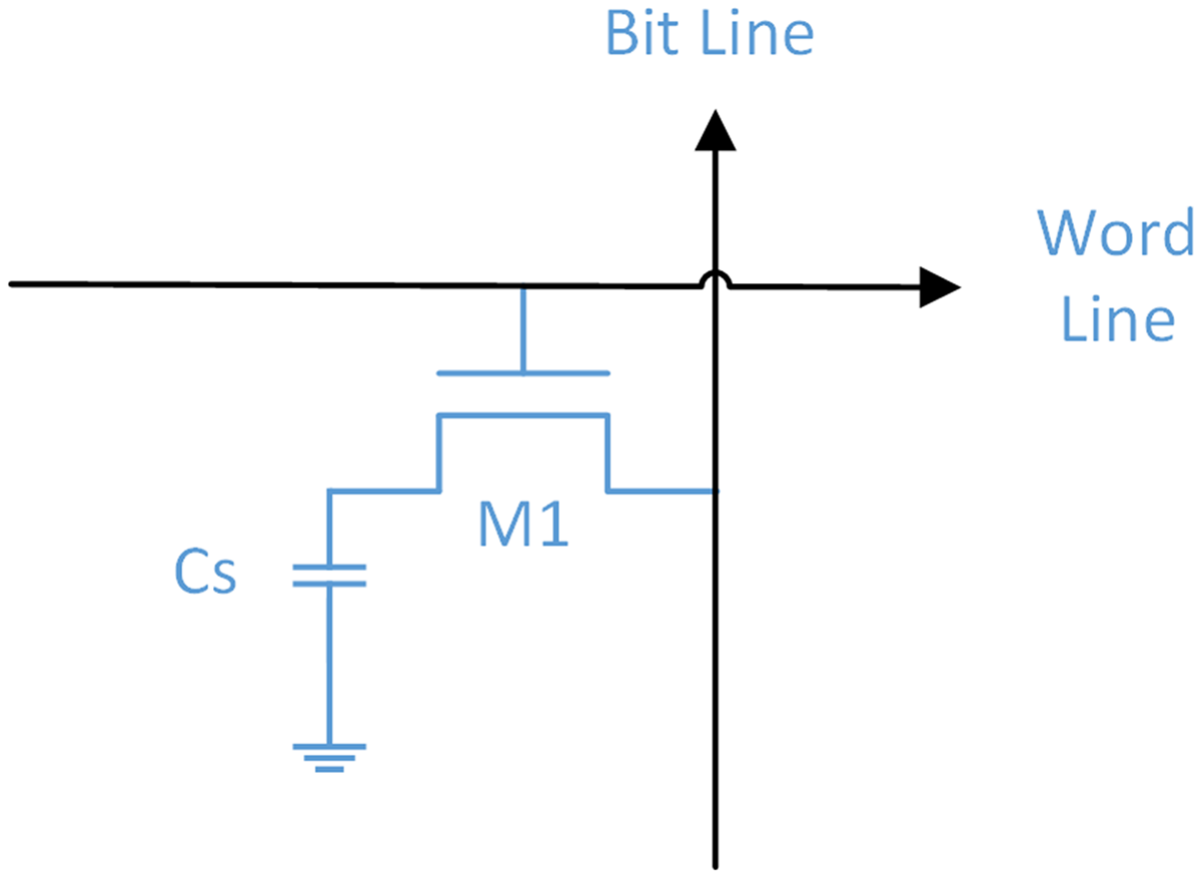
Conventional structure of the DRAM memory cell

In this research, we attempt to develop an engineered genetic circuit to fulfil a possible genetic dynamic random access memory (DRAM) for the future bio‐computer. Different from the electrical system using voltage and current transmission based on the Ohm's law, the biological system uses transcription and translation of mRNA and protein and creates output via the protein concentration. DRAM is a kind of semiconductor memory invented in [17]; it has been widely used as memory in modern computers at present. A typical DRAM memory cell consists of a capacitor $C_{s}$ and a transistor M1; the logic statuses 1 and 0 can be implemented by charging or discharging the capacitor (see Fig. 1) with the transistor controlling voltage transmission when accepting a sufficient voltage level over cut‐in voltage of the gate [18]. For the reading cycle, a sense amplifier is used to detect the unrecognised signal and convert it into the understandable logic level [19].

We present here a possible biological DRAM for the future bio‐computer. We use DNA transcription and mRNA translation to represent the equivalent DRAM mathematical model, and replace the sense amplifier with a genetic toggle switch because of its bistable characteristics. The proposed scheme is shown to be effective via *in silico* experiments.

## 2 Background

### 2.1 Operations of DRAM [17, 18]

DRAM is a kind of semiconductor memory and one of the main sections in the personal computer. Initially, a basic memory cell involves three transistors and one capacitor for data reading and storage. Nowadays, the commonly used DRAM employs the advanced structure of only one transistor and one capacitor. The DRAM memory cell uses an access transistor to control the data transmitted to the memory kernel and uses a capacitor to charge or discharge. It can be explained in more details in the follows.

**Fig. 2:**
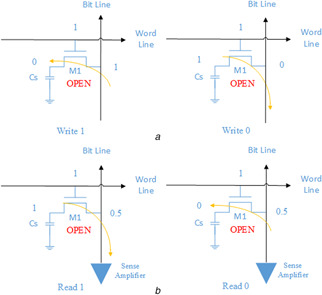
Operational steps of the DRAM memory cell

For data writing, the control unit of CPU imposes a high voltage to the word line, which activates the access transistor and turns on the gate. The voltage on the bitline transmits via the transistor to charge the capacitor or makes the voltage stored at the capacitor transmit to the bitline. See Fig. 2 *a* for the illustration.

For data reading, the operation begins by applying the intermediate voltage between ‘*H* ’ and ‘*L* ’. It then drives the high voltage to the word line causing the transistor to conduct. The voltage on the bitline is added to the capacitor (situation: stored 0) or added from the capacitor to the bitline (situation: stored 1). During transmission, the voltage on the bitline will be slightly increased or decreased while compared with the half value of the sum of ‘*H* ’ and ‘*L* ’ signals. The value, e.g. 0.45 or 0.55, detected by the sense amplifier will be amplified to represent an appropriate logic level. At the end of the read cycle, the control unit changes the word line status from *H* to *L* to close the transistor and ends the read operation, as shown in Fig. 2 *b*.

### 2.2 Model

**Fig. 3:**
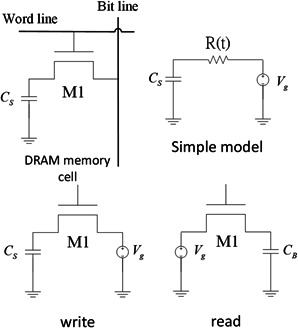
*Simplified model of DRAM* [17]

To establish the function of a memory cell of DRAM for the gene circuit, we start by considering a simplified model proposed in [20] that consists of a capacitor and a resistor to characterise the function of data writing and reading, as illustrated in Fig. 3.

For the writing operation, the bitline voltage is pre‐charged to the normalised level ‘0’ or the normalised level ‘1’, and then the normalised level ‘1’ is applied to the word line after a transient time period. The high level signal on the word line will turn on an *n* ‐channel transistor such that the bitline voltage is stored in the capacitor $C_{s}$. When the capacitor is fully charged, the word line voltage will be changed to $V_{s}=0$ to shut down the transistor, ending the writing process.

The following settings are applied in the simplified model:

(1) $$V_{g}(t)=V_{\text{DD}}(t),\;R(t)=r_{\text{ds}},\;C(t)=C_{s},\;\tau_{c}=r_{\text{ds}}C_{s}$$

where $V_{\text{DD}}$ is the supplying DC voltage, $V_{g}$ is the generator signal on the bitline, R(t) is the drain–source resistance of the transistor M1, and C(t) is the capacitor of the DRAM memory cell. In the writing mode, the transistor can be viewed as a resistor so that the circuit is considered as an RC charging circuit. By the Kirchhoff's voltage law and the dynamic response based on the RC circuit, the capacitor voltage $V_{c}$ is determined by

(2) $${\dot{V}}_{c}=\frac{1}{\tau_{c}}\left(V_{\text{DD}}-V_{c}\right)$$

That is

(3) $$V_{c}(t)=V_{\text{DD}}\left(1-e^{\left(-t/\tau_{c}\right)}\right)$$

For the reading operation, the bitline capacitor $C_{B}$ is brought to a pre‐charge voltage $V_{\text{Re}}$ before the transistor opens. After activating the access transistor, $C_{s}$ is connected to $C_{B}$ and the bitline resistor $R_{B}$. The model can be described as follows:

(4) Vg(t)=(VDD−ΔV−VRe)e(−t/τc)

where ΔV is the voltage change allowed by the operating and noise margins of the sense amplifier. Using the rudimentary model, the bitline voltage $V_{B}(t)$ can be obtained as

(5) VB(t)=VDD−ΔV−VReτcτB−τce(−t/τB)−e(−t/τc)

where the bitline time constant $\tau_{B}=R_{B}C_{B},\;R(t)=R_{B},\;C(t)=C_{B}$.

### 2.3 Sense amplifier

**Fig. 4:**
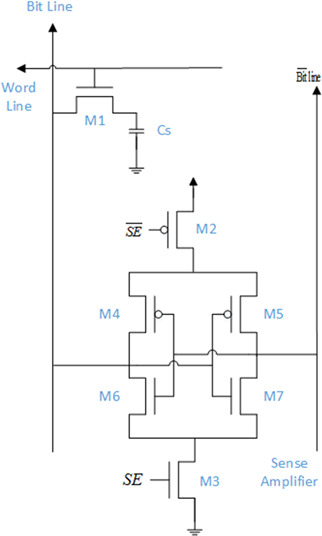
Structure of the sense amplifier

A sense amplifier is a key component of the circuitry on a semiconductor memory chip; it is a fundamental unit in the RAM design used in the read operation. The structure is shown in Fig. 4 where M2 and M3 are the access transistors, and M4–M7 form two cross‐coupled inverters. It is used to sense low power signal from the bitline and amplify this signal to a higher level one.

**Fig. 5:**
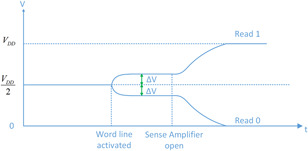
Operational waveform of the sense amplifier

In the read cycle, once the cut‐in voltage is established, the signal SE activates the transistors M2 and M3 $\left(\text{SE}=1,\;\bar{\text{SE}}=0\right)$. Due to positive feedback, higher level voltage pulls up the bitline voltage to $V_{\text{DD}}$ and other level voltages depending on the initial voltage signal on the bitline go towards to zero. The output will be pulled up to $V_{\text{DD}}$ if the signal is higher than $0.5\;V_{\text{DD}}$; on the other hand, the output will be drawn down to 0 when the input signal is lower than $0.5\;V_{\text{DD}}$. Fig. 5 shows the waveform of the sense amplifier operation.

Because the sense amplifier is too complicated to be duplicated in the biological sense, we propose to use a genetic toggle switch to fulfil the function of signal sensing and amplification.

## 3 Methods

### 3.1 Genetic toggle switch

A genetic toggle switch is used to implement the sense amplifier for the biological DRAM, which consists of two repressors and two promoters in its structure to fulfil the function. It holds the status of DRAM (by using the bistable feature). The following coupled first‐order differential model describes the behaviour of a genetic toggle switch [9, 21]:

(6) $$\begin{array}{rl} \frac{du}{dt} & =\frac{a_{1}}{1+v^{\beta }}-u,\\ \frac{dv}{dt} & =\frac{a_{2}}{1+u^{\gamma }}-v, \end{array}$$

where the state variables *u* and *v* denote, respectively, the concentrations of repressors 1 and 2, $a_{1}$ and $a_{2}$ denote, respectively, the effective rates of synthesis of repressors 1 and 2, β is the cooperativity of repression of promoter 2, and γ is the cooperativity of promoter 1.

**Fig. 6:**
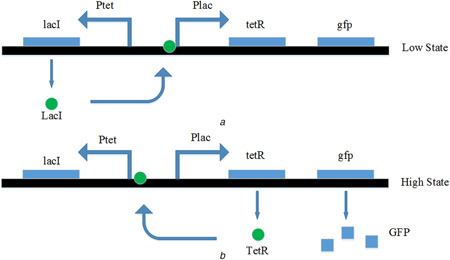
Bistable states of the genetic toggle switch

Considering the steady‐state response, there are two stable states referred to as the levels ‘high’ and ‘low’, respectively. As shown in Fig. 6 *a*, for the ‘low’ state, Ptet promoter transcripts to the lacI and is bound to the Plac promotor causing the repression of the transcription process from Plac to tetR and GFP. For the ‘high’ state in Fig. 6 *b*, the Plac promotor transcripts to the tetR, which is bound to the Ptet promotor causing the repression of transcription process from Ptet to lacI. Only ‘high’ level stimulates the fluorescent reporter protein (GFP), which is used as a reporter.

**Fig. 7:**
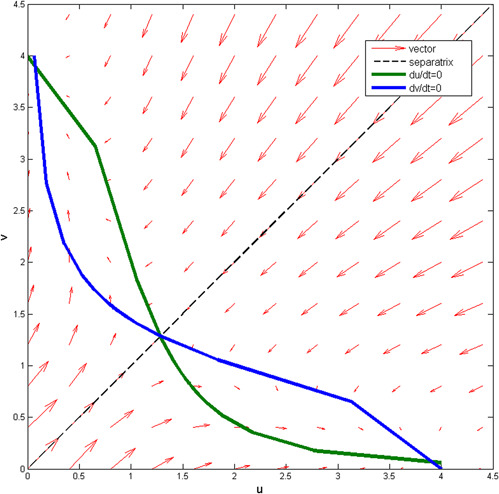
Nullclines and trajectories of the toggle switch

Fig. 7 shows the phase plane portraits of the genetic toggle switch model, in which the upper and lower lines are, respectively. The nullclines for $\dot{u}=0$ and $\dot{v}=0$, and the arrows denote the vector field indicating the direction of state behaviour at the corresponding time *t*. The dashed line separating two vector fields, depends on the initial condition of two states with each one converging to its steady state.

Here, we adopt the genetic toggle switch as the sense amplifier using its bistable characteristics. For the first situation, the initial state of (*u*, *v*) lying above the separating line will cause the steady concentrations of *u* and *v* settled to the state 1. Otherwise, *u* and *v* will be settled to the state 2 at the end when the initial state lies below the separating line.

For the design of the genetic DRAM reading operation, when the word line is activated, the signal level of the line level is expected to increase or decrease due to data transmission.

**Fig. 8:**
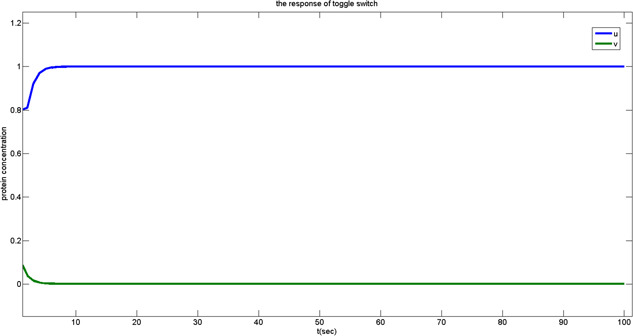
Output response of the toggle switch in situation 1

**Fig. 9:**
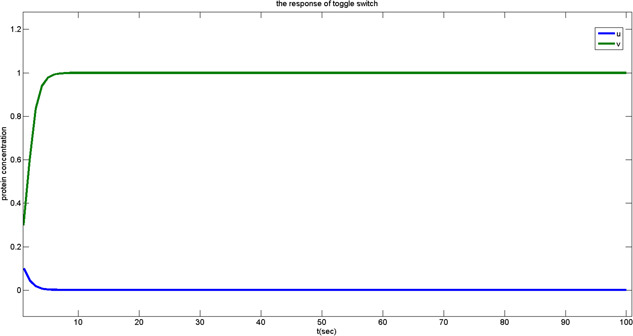
Output response of the toggle switch in situation 2

For the situation 1, the data stored is referred to ‘1’. In the electrical circuit, after the transistor turns on, the voltage rises from $0.5\;V_{\text{DD}}$ to $0.5\;V_{\text{DD}}+\Delta V$. It is equivalent to the biochemical reaction that the protein concentration increases during the processes of transcription and translation in the biological circuit. The response in Fig. 8 corresponds to the state located under the separating line in Fig. 9. After the genetic toggle switch works, the output concentration of *u* arises to ‘1’.

For the situation 2, the data stored is referred to ‘0’. After the transistor turns on, the bitline voltage starts to charge the memory cell, drawing the bitline voltage down from $0.5\;V_{\text{DD}}$ to $0.5\;V_{\text{DD}}-\Delta V$. This is equivalent to the biochemical reaction that the protein concentration decreases. It corresponds to the state located above the separating line in Fig. 7. The output concentration of *u* decreases to 0 after the genetic toggle switch activates. See Fig. 9 for the transient response.

### 3.2 Dynamic model of the genetic DRAM

We now turn to implement the genetic DRAM. Consider first the general model of the biological reaction. The typical dynamic model including a non‐linear Hill function can be described by [8]

(7) $$\begin{array}{rl} {\dot{m}}_{i} & =-m_{i}+\frac{\alpha }{\left(1+p_{j}^{n}\right)}+\alpha_{0},\\ {\dot{p}}_{i} & =-\beta (p_{i}-m_{i}), \end{array}$$

where *p* is the repressor protein concentration and *m* is their corresponding mRNA concentration, β is the ratio of the protein decay rate to the mRNA decay rate, α is the effective rate of synthesis of repressors, $\alpha_{0}$ is the number of protein copies produced per cell with saturating repressor level, respectively, and *n* is a Hill coefficient. Because the half‐life of mRNA is comparatively shorter than the corresponding protein, the equation can be simplified to

(8) $${\dot{p}}_{i}=-\beta p_{i}+\frac{\alpha \beta }{\left(1+p_{j}^{n}\right)}+\alpha_{0}\beta$$

Design of the DRAM memory cell can be realised by comparing its dynamic model with the dynamic protein translation. We convert the voltage in the electrical circuit in the biological sense of the protein concentration and select the appropriate Hill coefficients to obtain the write and read models.

In the write operation, we set $p_{j}$ to zero and the equation can be described as

(9) $${\dot{p}}_{i}=-\beta p_{i}+(\alpha +\alpha_{0})\beta$$

**Fig. 10:**
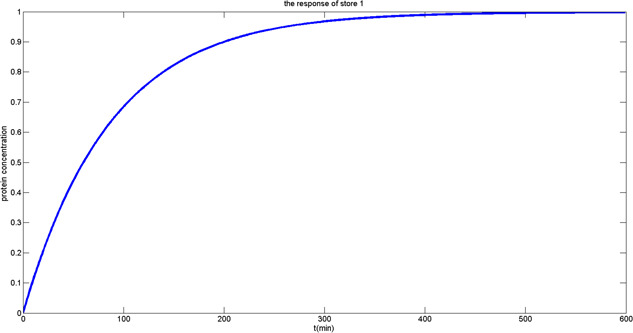
Output response of write ‘1’

**Fig. 11:**
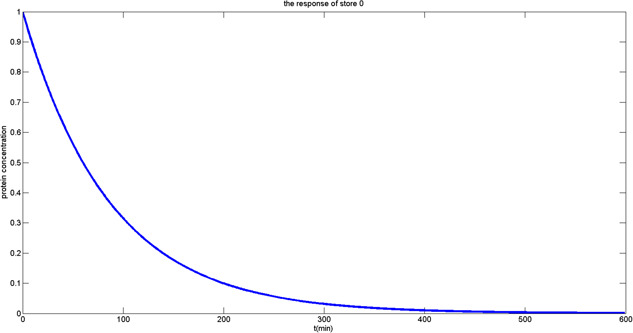
Output response of write ‘0’

Comparing the electrical model (2) to the biological model (9), it is quite clear that *p* represents the protein concentration stored in the memory cell, and $\alpha +\alpha_{0}$ is the eventual value stored. Equation (9) describes the dynamic behaviour of protein like an RC charging circuit, after a period of time, the voltage across the capacitor will essentially be equal to the bitline voltage. The growth and time course of the normalised protein concentration show the situation of storing data 1 to the genetic DRAM memory cell; on the other hand, reduction of the normalised protein concentration to zero is referred to the situation of storing data 0. Figs. 10 and 11 display dynamic responses of writing 1 and 0 when initial protein concentration is, respectively, 0 and 1.

For the read operation, the memory content is transmitted to the bitline depending on data stored within the genetic DRAM described by (8). For biochemical reaction, proteins are produced during the translation process. We use repressor gene to inhibit transcription, thus one can ignore the capacitor on the bitline, and modify the model to a simple RC charging circuit. In the biological equation, since $\alpha_{0}$ is usually zero for the stable state, we select the values $p_{j}$ and *n* to let the stable protein concentration to reach the steady state of $p_{i}=0.5P_{\max}$. The equation can be described as

$$\begin{array}{rl} & {\dot{p}}_{i}=-\beta p_{i}+\frac{\alpha \beta }{\left(1+p_{j}^{n}\right)}+\alpha_{0}\beta \\ & \left(1+p_{j}^{n}\right)=2\frac{\alpha }{P_{\max}} \end{array}$$

**Fig. 12:**
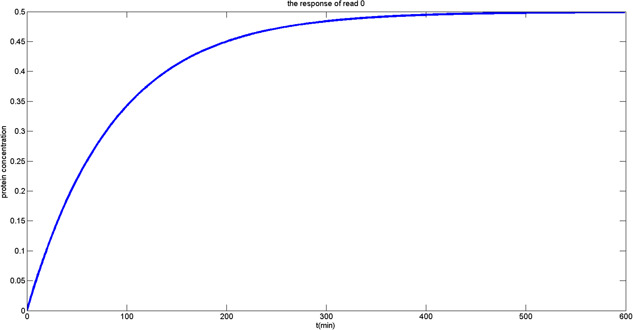
Output response of reading ‘0’

**Fig. 13:**
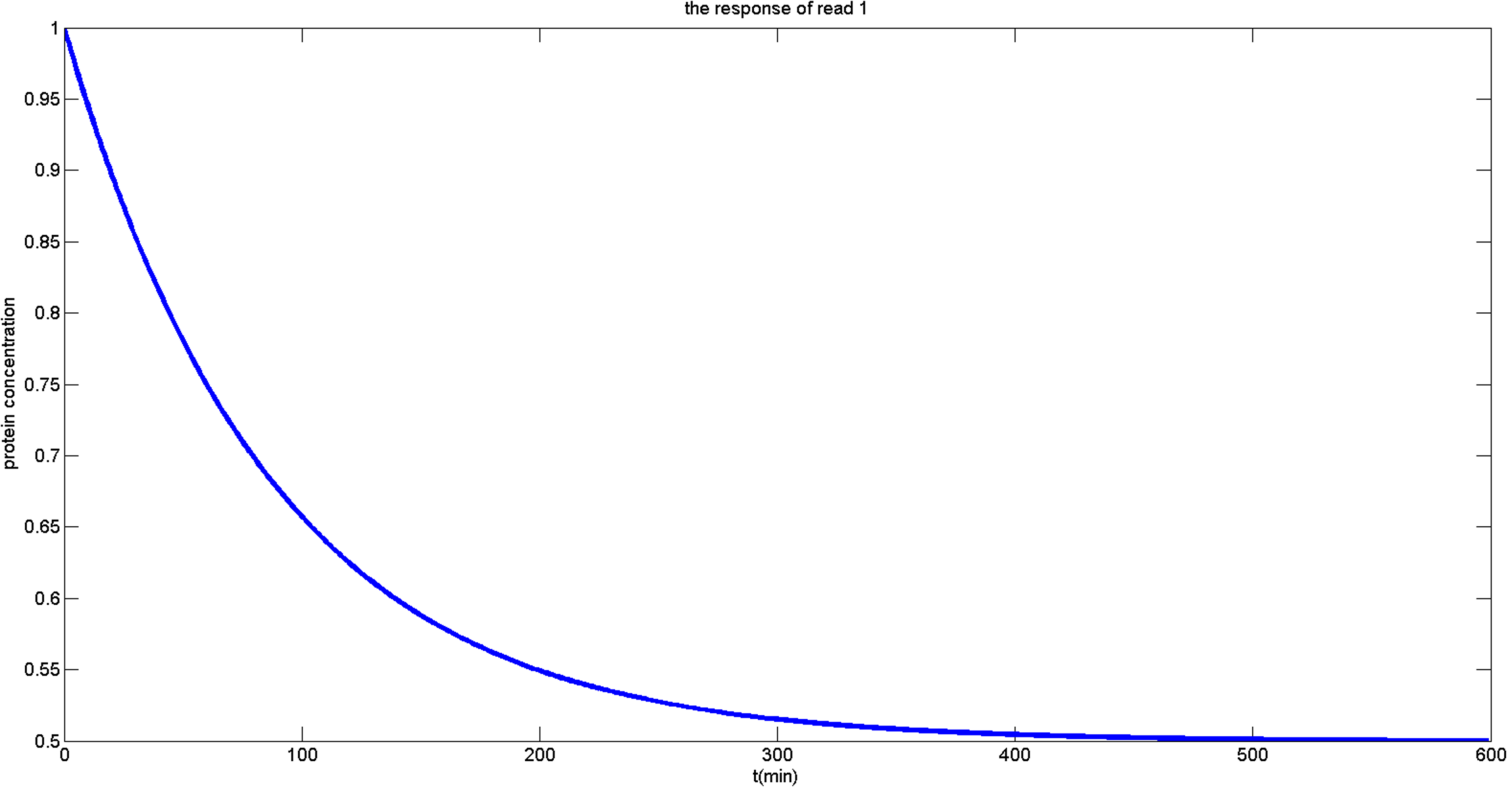
Output response of reading ‘1’

Fig. 12 shows the situation when 0 is stored in the memory cell. After the transcription and translation processes are activated, the gene starts to synthesise protein so that the protein concentration in the memory cell gradually increases from 0 to $0.5P_{\max}$. Fig. 13 describes the situation of the genetic DRAM when 1 is stored. In the biological reaction, the reduction of the normalised protein concentration corresponds to the interaction that repressor protein is bound to the promoter leading to the reduction of the normalised protein concentration from 1 to $0.5P_{\max}$.

As stated in [9], the structure of the toggle switch considered in Fig. 6 does not require any specialised promoters such as Pr/Prm promoter or bacteriophage. Bistability is possible with any set of promoters and repressors. Bistability is theoretically possible with a single, autocatalytic promoter. However, it is less robust and more difficult to be experimentally tuned. As for the memory cell, any protein with its dynamic characteristics that can be described by the Hill function (7) can be used for the current purpose. For example, considering the reduced‐order Hill equation model described by (8), it is used to mimic the dynamics behaviour of a DRAM cell described by the simplified model shown in Fig. 2. Actually, this is an RC circuit whose dynamic behaviour is just like the Hill equation (8). For example, for the write process in Fig. 3, the RC charging circuit is given by

$${\dot{V}}_{c}=-\frac{1}{RC_{s}}V_{c}+\frac{1}{RC_{s}}V_{g}$$

Comparing it to the Hill equation model (8), we have the relationship that $V_{c}$ corresponds to the protein concentration *p*, the time constant $RC_{s}$ corresponds to the term 1/β, and the bitline voltage $V_{g}$ corresponds the value of $\alpha +\alpha_{0}$. Analogous analysis can be established for the reading process. Therefore, one would expect similar dynamic behaviour of a genetic memory cell as expected in the electric circuit.

**Fig. 14:**
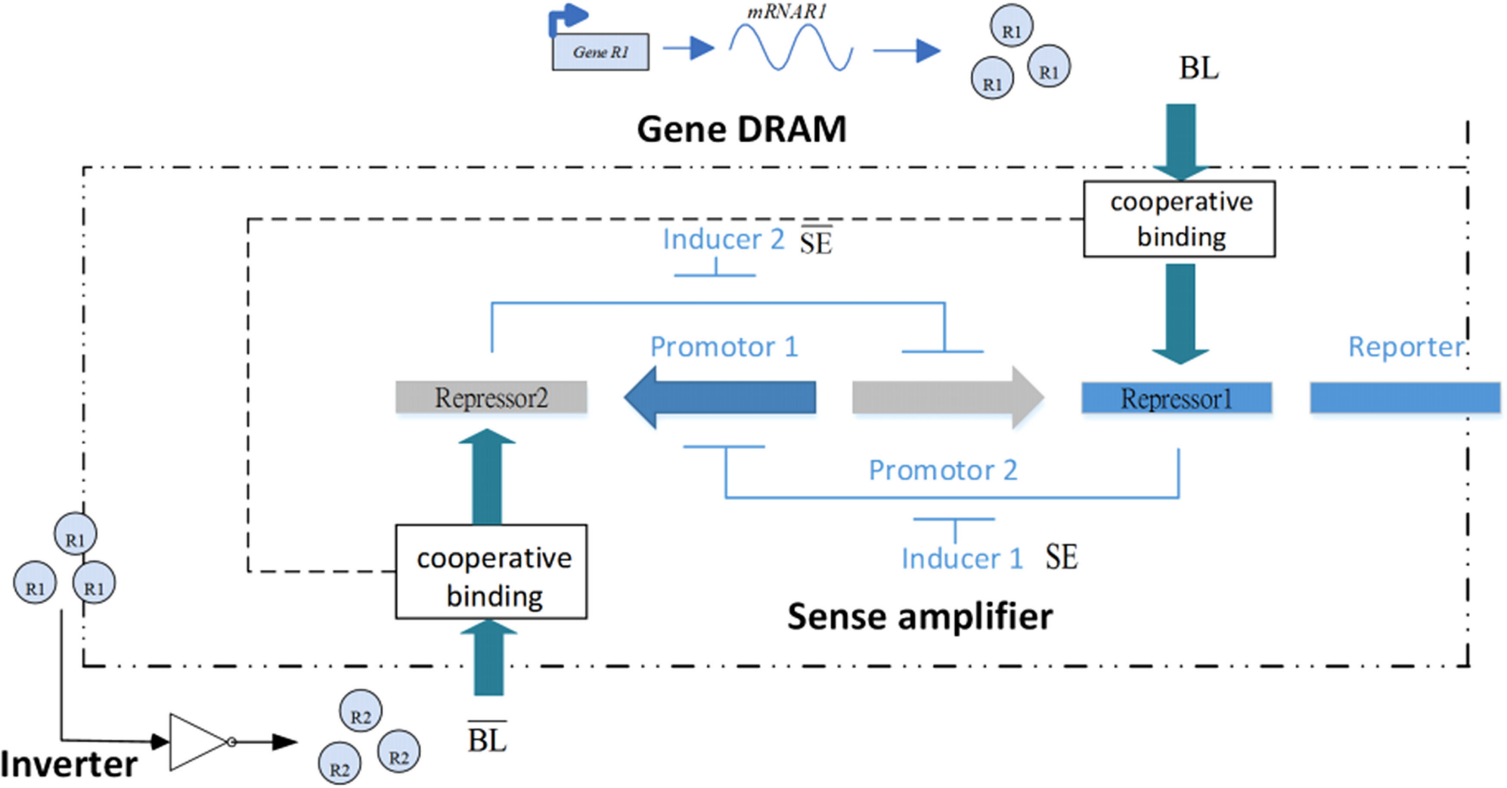
Architecture of the fundamental genetic DRAM for data read

The structure of the fundamental genetic DRAM for data read can be shown in Fig. 14, where $R_{1}$ denote the protein concentration of (8) and $R_{2}$ is the protein generated by an inverter with its input being $R_{1}$. The sense amplifier is activated by adding the inducer SE. The inverter can be realised via the available module developed in [5] or [10]. The function of the sense amplifier is described in Section 3.1.

## 4 Simulation results

**Fig. 15:**
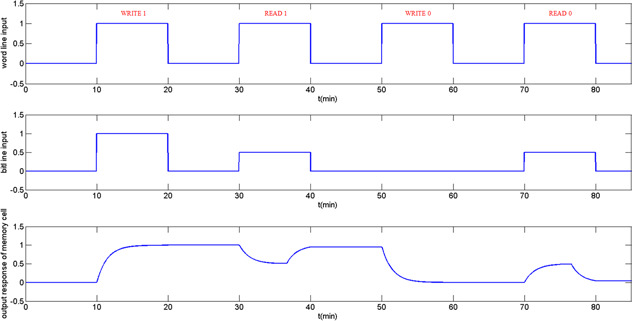
Sequence of operational cycles of DRAM

Fig. 15 demonstrates write–read operation of a one‐bit DRAM cell for normalised data ‘1’ and ‘0’. The time interval from t=10 to 20 describes the process of writing 1. Both of word and bitlines are promoted to high level which activates the reaction process. The protein concentration arises to logic ‘*H* ’ gradually after activation by the input stimulation.

The time interval from t=30 to 40 describes the process of reading 1 where the protein concentration at bitline was boosted to the normalised level 0.5. The read cycle starts when logic ‘*H* ’ applies to the word line, after the process, the protein concentration in the memory cell gradually decreases to $0.5P_{\max}+\Delta P$. The protein concentration stored will be pulled up to logic ‘H’ after connecting to the genetic toggle switch.

The time interval from t=50 to 60 describes the process of writing 0, which is similar to writing 1, but it changes the normalised bit level from 1 to 0. After the process reacted, the protein concentration stored in the memory cell declines from logic ‘*H* ’ to ‘*L* ’. A mechanism is added to turn off the word line terminating the reading progress after the reaction finished.

From t=70 to 80, data transmission between the memory cell and bitline makes the protein concentration in the memory cell increase to $0.5P_{\max}-\Delta P$. Similar to the reading 1 operation, after connecting to the genetic toggle switch, the protein concentration stored in the memory cell is drawn to logic ‘*L* ’.

As a concluding remark, the process in the biological system does not react as fast as that of voltage transmission in the electric circuit because it takes longer time to converge. In this simulation study, it shows a genetic DRAM operation cycle similar to that of the DRAM in electrical circuits. It correctly accomplishes the read/write operations of one‐bit memory cell.

## 5 Discussions and conclusions

This paper presents an idea of implementing a genetic DRAM memory cell which might be useful in the realisation of future bio‐computer. By steady‐state analysis, the model has characterised and confirmed the dynamic behaviour of the memory cell. The memory cell is realised by a standard gene regulatory unit. Moreover, the sense amplifier is realised by a genetic toggle switch to control charge or discharge of the capacitor which implements the function of data read or write of the memory cell.

So far, our preliminary study only focuses on the 1‐bit genetic DRAM. However, to fulfil the fundamental function of a primitive bio‐computer, a one‐bit memory cell is definitely not enough. Extension of the one bit DRAM to a complete memory module should involve far more than just one cell.

The 1‐bit genetic DRAM gene circuit considered in this research is simply constituted by the least number of gene components. When the module size increases with the need of more bits, the systems cannot just be placed in a cell without inducing negative effects on the well‐being of the cell. This could be resolved by distributing the circuits among a group of insulating cells or adding a different strong terminator with sufficiently diverse sequences to avoid homologous recombination. However, the problem of cell communication and synchronisation must be tackled. In addition, gene networks are generally suffered from intrinsic noises caused by molecular birth and death and extrinsic noises due to environmental perturbations [22]. As a result, the dynamical variation of concentrations may cause erroneous responses of the systems. It is possible to select appropriate regulatory parameters via the optimisation process to tackle some of the issues, however, it would need further investigation to substantially deal with these problems.
